# Generation Dynamics of Broadband Extreme Ultraviolet Vortex Beams

**DOI:** 10.1021/acsphotonics.4c02516

**Published:** 2025-02-28

**Authors:** Antonios Pelekanidis, Fengling Zhang, Kjeld S. E. Eikema, Stefan Witte

**Affiliations:** † Advanced Research Center for Nanolithography, Science Park 106, 1098 XG Amsterdam, The Netherlands; ‡ Department of Physics and Astronomy, Vrije Universiteit, De Boelelaan 1105, 1081 HV Amsterdam, The Netherlands

**Keywords:** high-harmonic generation, extreme ultraviolet, structured light, orbital angular momentum, wavefront sensing, ptychography

## Abstract

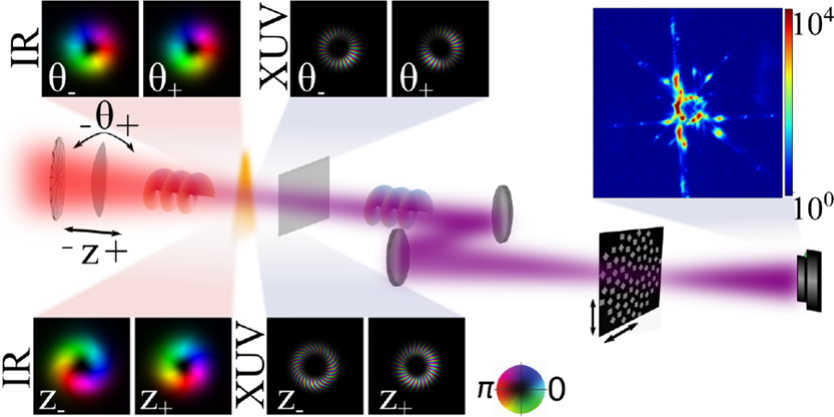

Light beams carrying orbital angular momentum (OAM) can be generated in the extreme ultraviolet and soft X-ray spectra by means of high harmonic generation (HHG). In HHG, phase properties of the drive laser, such as curvature, aberrations, and topological charge, are upconverted to the harmonic beams and coherently added to the inherent dipole phase. The strong nonlinearity of the HHG process, combined with the rapid phase variations corresponding to large OAM values in these vortex beams, leads to a high sensitivity to small variations in the driving field. However, a study of the generation dynamics via an accurate reconstruction of multiwavelength OAM beams is challenging. Here we show full complex field measurements of multiple individual harmonics of the HHG vortex beams. By using spectrally resolved ptychographic wavefront sensing, we retrieve the high-resolution amplitude and phase profiles for harmonics 23 to 29 in parallel, enabling detailed multiwavelength beam reconstructions. We study the influence of generation conditions and drive laser aberrations on the resulting vortex fields by comparing measured fields to numerical simulations and retrieving the propagation conditions around the focus and the OAM content of the beams. Specifically, we find that the multimodal content of such vortex beams can significantly influence the propagation and field distributions in the focal region. Such a beam propagation analysis allows a prediction of the resulting attosecond pulse trains and associated attosecond light springs that can be generated under realistic driving conditions.

## Introduction

Light beams carrying orbital angular momentum (OAM) have been of increased interest in recent years and have been proposed for various applications,^1,2^ including super-resolution microscopy,^3−6^ coherent diffractive imaging,^7,8^ optical trapping,^9,10^ communications,^11−13^ and quantum entanglement.^14,15^ OAM beams have a helical wavefront that is mathematically described as an azimuthal phase ramp e^*il*ϕ^, where *l* is the topological charge.^16^ OAM light beams with controlled properties have been demonstrated for different applications in a wide spectrum ranging from infrared to X-ray.^17,18^ Especially in the extreme ultraviolet (XUV) and soft-X-ray regime, OAM beams have been extensively studied theoretically,^19−24^ and demonstrated experimentally^7,25−37^ by means of high harmonic generation (HHG).^38−42^ For HHG OAM beams, the topological charge of the drive laser *l*_1_ is upconverted to *ql*_1_, where *q* is the harmonic order of each harmonic. The harmonic-order-proportional topological charge leads to the generation of spatiotemporal attosecond light springs.^19,43^

HHG is a fully coherent process; therefore, we can control the properties of the generated XUV and soft-X-ray beams via the drive laser. Thanks to the development of high-power ultrafast commercial lasers that render HHG broadly accessible, there has been extensive research on the configurability of HHG OAM beams. Specifically, earlier studies have demonstrated that we can achieve control over the resulting OAM of the XUV OAM beam by combining drive lasers with different topological charges^26,29,30,36^ and polarizations.^33,37^ It has also been shown that a drive laser with a minor impurity of its main OAM mode will lead to a large distribution of the HHG OAM modes.^21,34,35^ Such an impurity can be caused for instance by astigmatism and other aberrations in the drive laser^34^ or by fabrication limitations of the spiral phase plate (SPP) that is inserted in the drive beam in order to convert a Gaussian beam into an OAM beam.^35^

Moreover, as HHG is a coherent process, phase matching is required in order to maximize the yield. Experimental parameters such as gas pressure, laser intensity, focus position, and interaction length contribute to phase matching.^44−46^ Phase matching conditions vary for the different quantum paths, namely the short and the long electron trajectory, with the short trajectory usually having easier phase matching conditions.^32^ For OAM beams, specific studies revealed the conditions that lead to improved phase matching for HHG with a drive laser of topological charge equal to 1.^24,32,47^ All studies confirm that short trajectory harmonic emission has a longer coherence length and hence a higher HHG yield, and phase matching is better when the gas medium is behind the drive laser focus.

The wavefront of an emitted harmonic beam with harmonic order *q* can be modeled microscopically as Φ_*q*_ = *q*ϕ + Φ_*i*_, where ϕ is the wavefront of the drive laser at the generation plane and Φ_*i*_ is the dipole phase for short (*i* = *s*) or long (*i* = *l*) trajectory.^48−50^ This model has been used to predict the high harmonic wavefronts for perfect single OAM mode driving beams^19,20,22−24^ but also for driving beams with impurities in the dominant OAM mode.^21,35^ The studies assuming a pure OAM mode in the drive laser showed that the short-trajectory harmonic emission leads to a ring-shaped far-field intensity profile with similar divergence for all emitted harmonics.^19,23^ The properties of these beams are quite constant with respect to the distance between the drive laser focus and gas medium.^20,22^ However, the long-trajectory harmonic emission has a more diverse behavior as a function of laser focus-gas distance.^20,22^

HHG OAM beams have been studied experimentally with intensity-based measurements^27,28,47^ or with single-wavelength wavefront sensors.^31,34,35^ However, given the significant wavelength dependence of the dipole phase and extreme sensitivity to drive laser parameters, an accurate assessment of a broadband HHG OAM beam requires a full characterization of all harmonics in terms of amplitude and phase information.

In this paper, we use spectrally resolved ptychographic wavefront sensing (PWFS) measurements^51,52^ to retrieve complex fields of HHG vortex beams at multiple individual harmonics in parallel. By performing such measurements for varying drive laser parameters, we can quantify their influence on the generated HHG OAM beams. From the measured high-resolution field information, we then reconstruct the propagation behavior around the focus and the OAM modal content, which are found to depend on the combined effect of the dipole phase and the drive laser wavefront aberrations.

## Materials and Methods

### Experimental Design and Setup

To test the generation dynamics, we designed a series of experiments in which we performed high-resolution PWFS measurements on high harmonic beams while varying drive laser properties at the generation plane by adjusting the final focusing lens. Specifically, we vary the lens position along the beam propagation direction, thus changing the relative position between the beam waist and the gas jet as well as the lens tilt angle to introduce controlled amounts of astigmatism in the fundamental beam.

Our PWFS approach^52,53^ is based on the coherent diffractive imaging concept of ptychography,^51,54^ which reconstructs complex field information on an object and the incident illumination profile. By using optimized image masks, PWFS is capable of robust multiwavelength XUV wavefront sensing of individual harmonics,^53,55^ even for complex fields with high OAM.^8^ In addition to the HHG wavefront sensing, we perform PWFS measurements on the fundamental beam for all demonstrated generation conditions so that we can compare the measured HHG wavefronts with theory predictions from a single atom response model, which is described in the next section.

**Figure 1:**
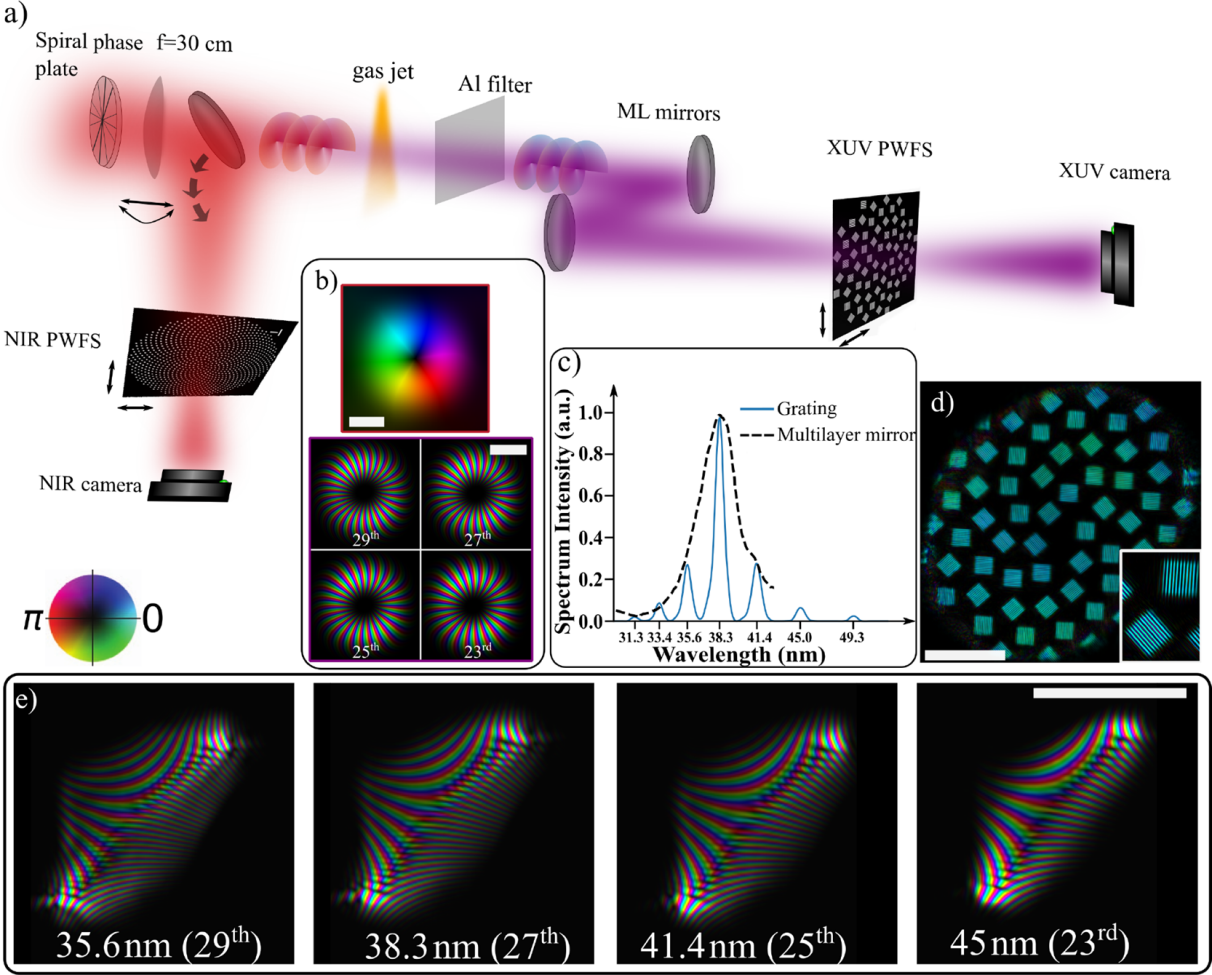
Experimental setup. (a) A NIR laser with topological charge equal to 1 is focused to a gas jet to generate HHG. The high harmonics propagate to a pair of multilayer mirrors that focus the beam in the vicinity of the ptychographic wavefront sensor plane. An auxiliary beam path in front of the vacuum part is used for ptychographic wavefront sensing of the fundamental beam. (b) Example of reconstructed NIR beam (top) and modeled high harmonic wavefronts at the gas jet plane (bottom) for that particular NIR beam. (c) High harmonic spectrum behind the multilayer mirrors measured through diffraction from a grating, indicated by the solid line. The dashed line shows the reflectivity of the multilayer mirror pair. (d) Ptychographic reconstruction of the wavefront sensor. Inset shows the zoomed-in area of the object. (e) Wavefronts of harmonics 23 to 29 at measurement plane. In (b–e), brightness indicates the amplitude and hue indicates the phase, according to the colormap in the left of the figure. Scale bars in all figures correspond to 50 μm.

The complete setup is shown in Figure 1a. An ultrafast ytterbium-based NIR laser system (Pharos from Light Conversion) drives the HHG. The laser system delivers 2 mJ pulses at a center wavelength of 1030 nm with a pulse duration of 170 fs and a repetition rate of 1 kHz. For efficient HHG, the pulses are compressed by a home-built pulse compression system to a duration ≈45 fs with an average power of 1.5 W.^56^ The NIR beam, with an fwhm diameter equal to 6 mm, is sent through a spiral phase plate (Vortex Photonics V-1064-20-1) giving a topological charge equal to 1 at a nominal wavelength of 1064 nm and is subsequently focused by a 300 mm lens into a jet with 1 mm diameter, filled with argon at a backing pressure of 5 bar. Moreover, an iris with a diameter equal to 7.7 mm clips the beam before the focusing lens in order to maximize the HHG flux.^57^

Behind the focusing lens, a flip mirror can send the beam to an auxiliary beam path, where we perform the PWFS of the fundamental beam at a plane that corresponds to the vicinity of the gas jet. The NIR wavefront sensor is a binary object with 4 μm square holes arranged in a golden spiral configuration with a total width of 400 μm. The diffraction patterns from the NIR wavefront sensing measurement are recorded by a CCD camera (AVT GT3400, 3384 × 2704 pixels, pixel size 3.69 μm, 14-bit dynamic range) placed 12.5 mm behind the wavefront sensor.

Within the main beam path, an aluminum filter blocks the drive laser and transmits the generated high harmonics, which are then focused by a pair of plane and curved (ROC = 500 mm) molybdenum/silicon multilayer mirrors (optiXfab GmbH) onto the wavefront sensor. The HHG spectrum, shown in Figure 1c, is shaped by the efficiency of the multilayer mirrors and includes seven wavelengths ranging from 31 to 50 nm. The wavefront sensor is a scaled version of the one proposed in a previous study^53^ and consists of 10 μm apertures with four orientations and 1 μm pitch gratings inside each aperture. An example of the ptychographic reconstruction of the object is shown in Figure 1d. A CCD camera (Andor Ikon-L 936SO, 2048 × 2048 pixels, pixel size 13.5 μm, 15-bit dynamic range) is placed approximately 107 mm behind the sample. The numerical aperture of the ptychographic setup is 0.129, leading to a diffraction-limited resolution of 148 nm for the central HHG wavelength of 38.3 nm.

### Single Atom Response Model

The reconstructed fundamental complex electric field can be used to estimate the high harmonic wavefronts. Here, we adopt the single atom response (SAR)^49,50^ which gives expressions for both the amplitude *A* and the phase Φ of each high harmonic *q* in the plateau region:
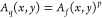
1
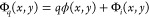
2
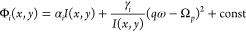
3In the above expressions, *A*_*f*_(*x*, *y*) and ϕ are the amplitude and phase of the fundamental beam at the generation plane, and Φ_*i*_ is the dipole phase. For the calculation of the spatial distribution of the dipole phase, *I* and ω are the intensity and frequency of the fundamental laser respectively, and Ω_*p*_ is a frequency associated with the ionization potential *I*_*P*_ of the gas Ω_*p*_ = *I*_*p*_/*ℏ*. The parameter values used to describe the amplitude and short-trajectory dipole phase are taken from,^50^ with a wavelength scaling for a 1030 nm drive laser wavelength where applicable: γ_s_ = 0.795 × 10^–18^ s^2^ W·cm^–2^, α_s_ = 0, and *p* = 4. This model for the dipole phase has been used in previous studies to describe chromatic aberration effects in HHG Gaussian beams and transfer of aberrations from the drive beam to the high harmonics, and was in agreement with experimental findings.^53,55^Figure 1b shows an example of the predicted HHG wavefronts for the four brightest harmonics (23rd to 29th harmonic), using the reconstructed drive laser information as input for the single atom response model.

### Ptychographic Reconstruction of High Harmonic Wavefronts

For the ptychographic wavefront sensing, we have placed the object on a translation stage that can move laterally to the beam (Smaract SLC-1730) and acquired a series of 218 diffraction patterns with the object moving in a concentric scan grid covering a field of view equal to 104 μm with an average step size equal to 6 μm. The ptychographic reconstructions are performed via Ptylab.py.^58^ The overlap factor, using the definition of the overlap presented in,^8^ is equal to 68%. The relatively low overlap factor given the complexity of the reconstruction problem with seven unknown structured wavefronts creates some uncertainties in terms of the reconstructed topological charges. Specifically, while the topological charge for every wavelength should be equal to the respective harmonic order when the drive laser’s topological charge is equal to 1, the reconstructed topological charges are in the range between harmonic order ±2. Examples of independently reconstructed beams from the same data set that exhibit different topological charges are shown and discussed in the Supporting Information. Therefore, the results shown in the following section are based on a constrained reconstruction process, such that the topological charge of each probe, calculated during the reconstruction process as ,^59^ is fixed according to the HHG upconversion rule for the topological charges. While the topological charge is a single value that is set by the physical properties of HHG, the OAM of the harmonics can be influenced by the generation conditions. As the nonlinear conversion process will influence the exact amplitude and phase profiles of each high harmonic wavefront, the harmonics can carry a distribution of OAM values with a central value that may deviate from the topological charge.^21,34,35^ The OAM distribution can then be calculated via an azimuthal Fourier transform (FT) of the complex electric field on a circular path along the beam^35^ or via a Laguerre–Gaussian decomposition.^34^

### Correction of Multilayer Mirror Effects on Wavefronts

The presence of the refocusing optics needs to be considered for the numerical propagation of the HHG beam between the generation plane and the wavefront sensor plane. The indicated angle of incidence for maximized reflectivity is 5°. For the plane mirror, we assume that there is perfect reflection and neglect any surface defects. However, oblique incidence on the curved mirror leads to astigmatism, as can be clearly observed in the example ptychographic reconstruction of the vortex probes in Figure 1e. The induced astigmatism to the wavefront is equal to , with η the incidence angle and *r* the radius of curvature of the mirror.^60^ In practice, the beam has a slight elevation angle, and the incidence angle can deviate from exactly 5° for alignment purposes. Therefore, the astigmatism contribution can only be estimated such that the astigmatism-corrected wavefronts have physically reasonable profiles at the focus, which is an image plane of the generation position. It is also convenient to perform amplitude comparisons between modeled and experimental results at the curved multilayer mirror plane, as the beam intensity profile has not been affected by the astigmatism induced by the curved multilayer mirror. Since the PWFS measurements give a high-resolution representation of both the amplitude and phase of the high harmonic wavefronts at the object plane, we can get beam information at the curved multilayer mirror plane via numerical propagation of every reconstructed wavefront assuming the paraxial approximation. The numerical propagation can be performed, for instance, with the scaled angular spectrum propagator.^61^

## Results and Discussion

### Dependence of the HHG Beam Profiles on the Drive Laser Properties

**Figure 2:**
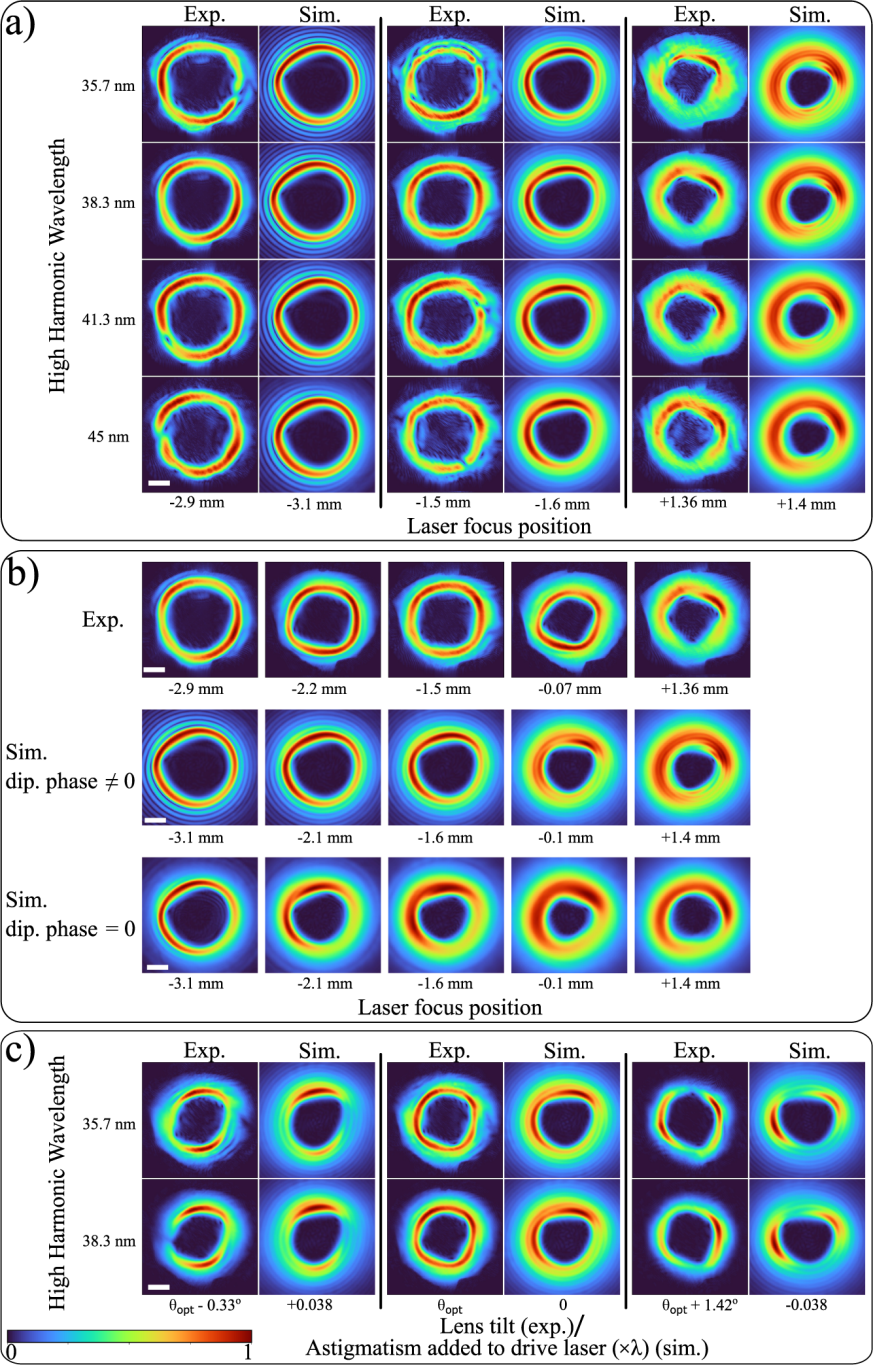
Reconstructed and modeled beam amplitudes for four harmonics ranging from 35.7 nm (29th harmonic) to 45 nm (23rd harmonic) at the curved multilayer mirror plane for various generation conditions. (a) Beam amplitudes for varying laser focus positions with respect to the gas medium. Positive (negative) laser focus positions mean that the laser focus is downstream (upstream) of the gas jet position. (b) Comparison of reconstructed and modeled beams for 27th harmonic with and without the dipole phase contribution in the model. (c) Beam amplitudes of harmonics 29 and 27 for varying tilt positions of the focusing lens. The beam profiles for lower harmonics are similar and shown in the Supporting Information, along with results for more lens tilt positions. The absolute laser focus positions for experimental data in (a) and (b) are approximate, based on the fit with the modeled results. Scale bars in all figures are equal to 3 mm.

First, we investigate the HHG beam amplitudes as a function of the laser focus position relative to the gas medium. The measured and reconstructed complex fields of harmonics 23 to 29 are numerically propagated back to the curved mirror plane and compared to simulations. In Figure 2a, we show the results for 3 example laser focus positions (−2.9, −1.5, and +1.36 mm), while results for more positions are shown in the Supporting Information. From Figure 2a, we observe the same trend with respect to the rim thickness as modeled in a previous report,^20^ with a single thick ring for the cases when the drive laser focus position is behind the gas medium, whereas the HHG beams exhibit a thinner rim of the main ring and secondary rings with larger diameter when the gas is on the divergent side of the drive laser. The secondary rings appear to be clipped due to an added aluminum foil that was intended to block leaking fundamental light through the flat multilayer mirror toward the camera, but still, the presence of these rings is clear, especially in the short wavelengths. In Figure 2a, we also show comparative results from expected far-field high harmonic beam profiles using the SAR model. The drive laser’s beam profile at the generation plane assumed in the model is a reconstructed beam from the auxiliary drive laser path shown in Figure 1a, which we numerically backpropagated to different distances along the focus, in order to simulate the generation conditions for varying laser focus positions. Due to small beam drifts between HHG and NIR wavefront sensing measurements, we numerically corrected some of the aberrations in the drive laser at the focusing lens plane before propagating to the respective generation plane and applying the SAR model. The Rayleigh length has been calculated to be approximately 6 mm, so the experiment covers a range of about half a Rayleigh length in both directions from the laser focus.

We find that the model captures the trend of the experimentally derived beam profiles’ behavior as a function of the laser focus position. For focus positions in front of the gas, we predict a thin bright ring and secondary rings with higher divergence, which are more profound for the short wavelengths. On the other hand, when the drive laser focus position is behind the gas, we predict a single ring with increased rim thickness. We should note here that these results consider only short electron trajectory contributions to the HHG, as long trajectories have very short phase matching length^32^ and their efficiency is considered negligible for our generation conditions. In previous work, secondary rings were predicted^20^ and observed^28^ for positive laser focus positions, and associated with long trajectories. The appearance of ring structures attributed to long trajectory emission has some analogy to experimental studies^62,63^ for Gaussian beams. It is likely that the shorter Rayleigh length and the longer medium used in our HHG setup result in phase-matching conditions that suppress the long trajectories.

The influence of the dipole phase of the short trajectory is illustrated in Figure 2b, where we compare far-field HHG profiles for different γ_s_ values in the SAR model for the 27th harmonic, with γ_s_ = 0.795 × 10^–18^ s^2^ W·cm^–2^ and γ_s_ = 0. From this figure, it can be seen that the secondary rings are caused by the dipole phase and subsequent beam propagation. For reference, in the Supporting Information, we present modeled HHG wavefronts that would correspond to long electron trajectories. For the specific drive wavelength, pulse duration, and pulse energy in our experiments, we observe that the wavefronts associated with long trajectories would diverge strongly and not be captured by the refocusing optics. In the Supporting Information, we also show far-field beam intensities from short trajectory contributions, when the drive beam is an ideal circularly symmetric nonaberrated vortex beam. In the case of this ideal drive beam, the far-field HHG beam profiles for varying drive laser focus positions with respect to the generation plane closely resemble other theoretical results,^20^ with secondary rings not present in the beam. Therefore, our conclusion is that the experimentally observed rings (Figure 2a) are caused by the combination of focusing geometry, fundamental beam profile, and dipole phase. Note that this conclusion does not exclude the possibility of forming additional rings in a geometry where long trajectories are generated more efficiently.

Similarly, in Figure 2c we compare experimental and model results for varying astigmatism of the drive laser. For these measurements, the distance between the gas jet and the nonaberrated laser focus was set to −0.54 mm. The astigmatism is adjusted by tilting the focusing lens in a controlled way in a range of 1.75° around the optimized tilt, denoted as θ_opt_. This optimized tilt angle leads to a polychromatic HHG beam at the camera with the most uniform intensity around the ring. We model the astigmatism by numerically adding astigmatism in the beam used to generate the simulated results (Figure 2a). We observe a close similarity of the beam amplitudes, with the doughnut-shape of the HHG beams transforming into two bright lobes either in the vertical or horizontal direction. Note that adding drive laser astigmatism of only 0.038λ already leads to the emergence of bright lobes in the HHG beams due to the strong nonlinearity of the upconversion to the high harmonics.

### Propagation of Vortex Beams around a Focus

As mentioned before, the phase of the HHG beams can be corrected at the curved multilayer mirror plane for each investigated generation condition if we numerically remove the astigmatism induced by the mirror due to the nonzero angle of incidence. Since the precise astigmatism of the mirror is not known with sufficient accuracy, we used fitted astigmatism coefficients for the results shown henceforth such that they maximize the OAM modal purity of the most circular and azimuthally homogeneous beam profile from the beam profiles of Figure 2a. This assumption implies that a beam with an amplitude profile that resembles a pure LG_*q*,0_ mode, with *q* the harmonic order, is more likely to have a phase that corresponds to a pure LG_*q*,0_ mode, too. We selected the 27th harmonic (38.3 nm) from the data set that corresponds to laser focus position −1.5 mm as the beam with maximized OAM purity, which gave astigmatism coefficients equal to α_1_ = 4.959*k*·10^–3^ and α_2_ = −4.934*k*·10^–3^, with *k* the wavenumber for the 27th harmonic and α_1_, α_2_ such that Φ_astigm correction_ = e^–*i*(α_1_ρ^2^cos(2ϕ)+α_2_ρ^2^sin(2ϕ))^, where ρ and ϕ are the polar coordinates. For reference, the expected astigmatism according to,^60^ for angle of incidence equal to 5° and a radius of curvature equal to 50 cm, is 7.62*k*·10^–3^(*x*^2^ – *y*^2^).

**Figure 3:**
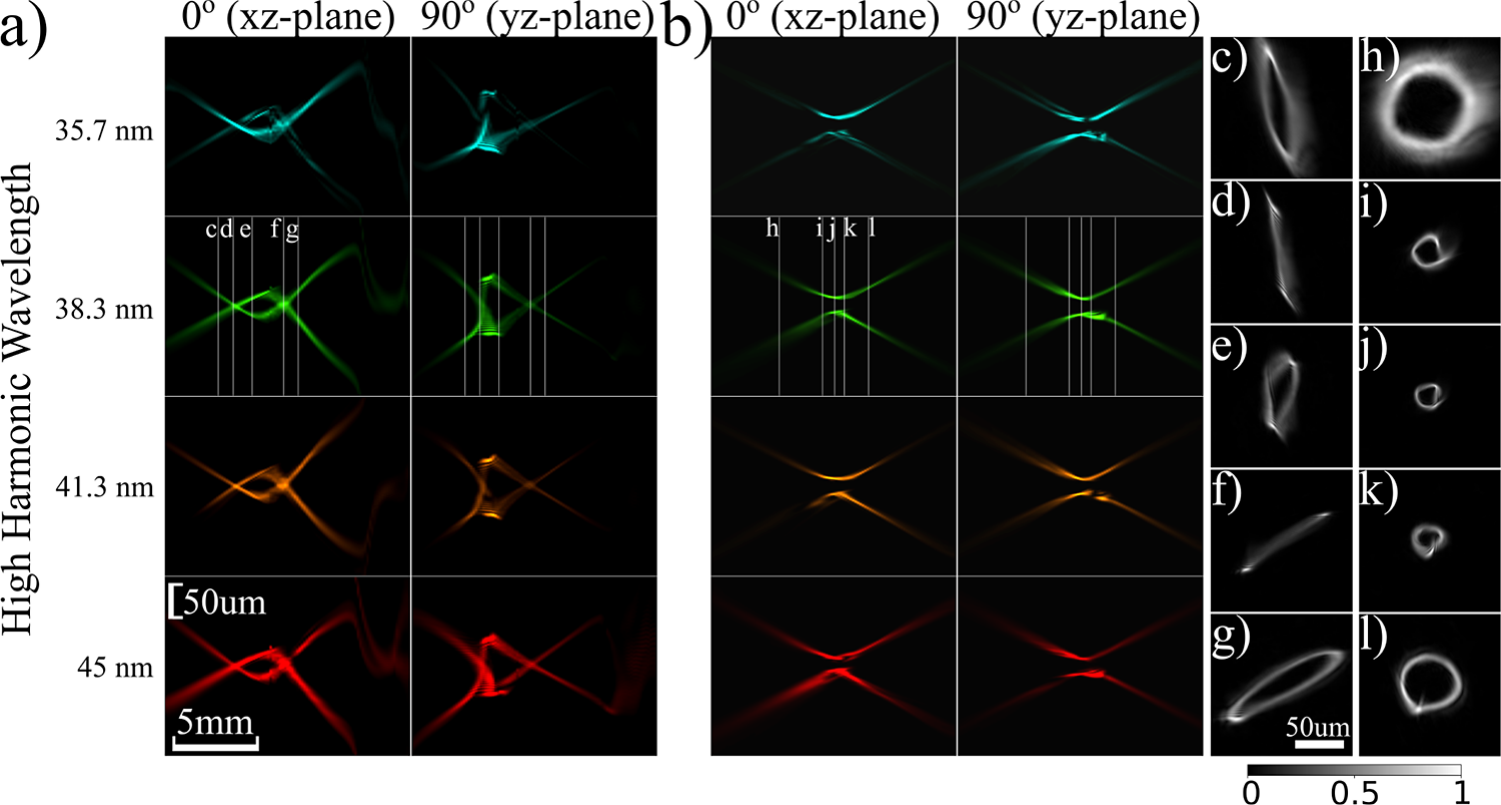
Propagation of harmonic beams along the focal plane after refocusing. (a, b) Cross sections along horizontal and vertical cuts for (a) the reconstructed wavefronts from the PWFS measurement, (b) the astigmatism-corrected beams at the curved multilayer mirror plane which are subsequently forward propagated around the focus, and (c–g) in-plane amplitude profiles of the 27th harmonic (38.3 nm) for planes denoted in the second row of (a). (h–l) the same as (c–g), for the astigmatism-corrected beams at planes denoted in (b). Scale bars are shared between (a, b) and (c–l).

In Figure 3, we show example propagation plots of the four reconstructed harmonic beams along the measurement plane without (Figure 3a) and with (Figure 3b) the astigmatism correction, starting from the reconstructed fields for the laser focus position at −1.5 mm. We show two different cross sections, the *xz* and *yz* planes, as well as 2D amplitude plots at selected planes. In the Supporting Information, we show propagation results across the diagonal cross sections as well, where we see similar behavior as in the *xz* and *yz* planes. Figure 3c–g) shows in-plane plots of the 27th harmonic at different planes along the focus position. The astigmatism from the spherical multilayer mirror leads to distorted beam profiles, where at some planes the doughnut shape has degenerated into a thin line (Figure 3d, f). After removal of the astigmatism term at the multilayer mirror plane and propagation again forward along the focus, all harmonic beams maintain the doughnut-shaped amplitude profile and can be refocused to a smaller spot size (Figure 3h–l). However, even for the numerically corrected beams for astigmatism from the spherical multilayer mirror, we observe in Figure 3h–l that there is a slight intensity variation across the beam as the beam propagates along the focus. This is an indication of a remaining OAM modal impurity, with the coherent superposition of the different OAM modes creating this intensity variation effect.

**Figure 4:**
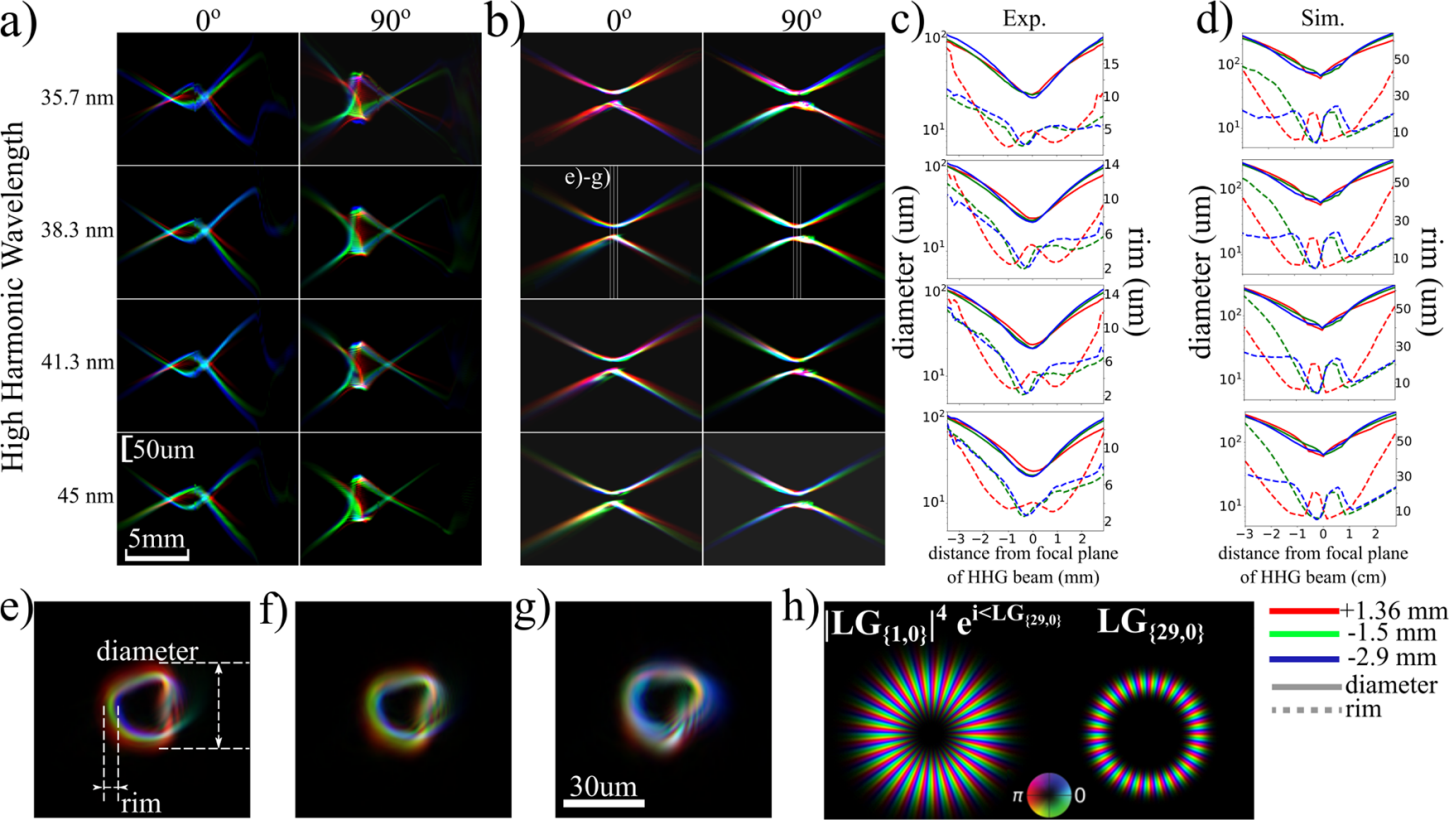
Propagation of harmonic beams along the focal plane after refocusing, overlapped for different laser focus positions. Red: + 1.36 mm, green: – 1.5 mm, blue: – 2.9 mm. (a, b) Cross-sections along horizontal and vertical cuts for (a) the reconstructed wavefronts from the PWFS measurement, (b) astigmatism-corrected beams at the curved multilayer mirror plane which are subsequently forward propagated around the focus, (c and d) experimental and model results for the variation of diameter and rim thickness, as defined in (e), of the astigmatism-corrected beams, for different wavelengths and generation conditions. In (a–d), all horizontal rows are aligned and correspond to the same wavelength. (e–g) Overlapped in-plane amplitude profiles of the 27th harmonic (38.3 nm) for planes denoted in the second row of (b). (h) Example of a 29th harmonic beam driven by a LG_1,0_ fundamental laser (left) compared to a LG_29,0_ mode (right). Brightness indicates the amplitude and hue indicates the phase. Scale bars in (a, b) and (e–g) are shared.

Figure 4 shows the propagated beams around the focal region for different laser focus positions. Propagation is performed directly with the retrieved fields (Figure 4a) and after numerical astigmatism removal (Figure 4b). While the retrieved field results do not require an assumption about mirror-induced astigmatism, the corrected fields provide better insight into the HHG beam properties in the vicinity of the generation plane. Figure 4e–g shows overlapped, for different laser focus positions, in-plane plots of the 27th harmonic at 3 planes around the focus. From these reconstructions, we find that the rim width and the ring diameter of the vortex beams are influenced by the laser focus position in different ways. These quantities are plotted in Figure 4e: the diameter is measured between intensity maxima, and the rim thickness is defined as the fwhm, both azimuthally averaged. The focal plane of the HHG beam is taken as the plane where the diameter is minimized. Using these two parameters to describe the OAM beams, we observe that the drive laser focus position affects both diameter and rim thickness, as shown in Figure 4c, d for the experiment and model, respectively. The model is based on the drive laser that was used to generate the amplitude results (Figure 2a).

A striking feature is the local increase in rim thickness near the focal plane, which is dependent on the generation conditions. The simulations are in agreement with the experiment and can qualitatively explain this feature. In the simulations, the peak in the rim thickness coincides with the generation plane. The amplitude of the HHG beams at the generation plane is modeled as the drive laser amplitude to the power 4 (which is typical in the strong-field approximation). In Figure 4h, we show a simplified simulation example to better explain the HHG beam behavior around the generation plane. Taking the drive laser as a pure LG_1,0_ mode, the 29th harmonic will have the amplitude and phase profile of the beam shown in the left panel of Figure 4h at the generation plane. Compared to a pure LG_29,0_ mode (right panel of Figure 4h), such a beam has a clear difference in the rim thickness. Therefore, the 29th harmonic should be described as a superposition of different radial modes LG_29,n_,^34^ which at the generation plane are coherently added to create a ring with increased thickness. However, as this beam propagates, the radial modes have different relative phases and are no longer added constructively. This effect causes the rim to become narrower initially, as it is mainly determined by the dominant LG_29,0_ mode until it starts expanding again due to propagation. An animated example of beam propagation along the focus is given in the Supporting Information, in which the diameter and rim variation are clearly visible. The effect of the dipole phase is a local divergent wavefront on the rim, which favors the expansion of the rim and partially counteracts the aforementioned effect. Further insight into the effect of the dipole phase is given in the Supporting Information, where we show modeled results excluding the contribution of the dipole phase.

The reasoning above also explains the position of this local maximum in the rim thickness relative to the HHG focal plane. The rim local maximum occurs at the generation plane, where the phase properties of the drive laser are transferred to the harmonic wavefronts. Therefore, for positive laser focus positions, the harmonic beams have a converging phase, and the focus is real and downstream of the generation plane, whereas for negative laser focus positions the harmonic beams diverge at the generation plane and have a virtual focus. Note that the scales are different between the experiment and the model because the experiment has been conducted at the (corrected) image plane with a demagnification ratio of 2.76 between the gas jet and the image plane.

The comparison between experimental and modeled results shown in Figure 4c,d can provide insight into the exact value of the nonperturbative scaling power *p* in eq 1, as *p* can vary depending on the generation conditions and other values have been proposed except *p* = 4.^20^ However, as the modeled results presented in Figure 4d have been calculated with the SAR model, a more quantitative comparison between experimental and simulated results can only be accurate for HHG configurations with a thin generation medium, where phase matching conditions can be excluded. In our experiments, we can safely consider the generation medium to be thin compared to the drive laser’s Rayleigh length, but still, such a quantitative comparison would likely require a more accurate modeling of the HHG process.^64^

**Figure 5:**
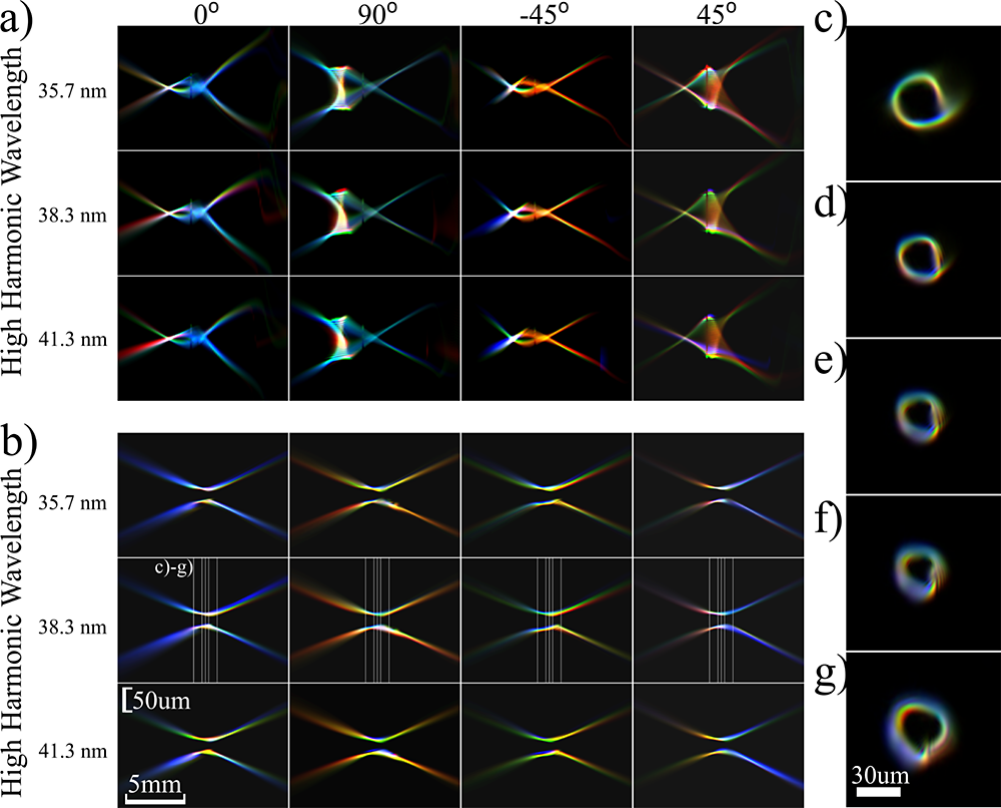
Propagation of harmonic beams along the focal plane after refocusing overlapped for different astigmatism levels of the drive laser. Lens tilt positions of red: θ_opt_ – 0.33°, green: θ_opt_, blue: θ_opt_ + 1.42°. (a, b) Cross sections along different cuts (horizontal, vertical, and diagonal) for (a) the reconstructed wavefronts from the PWFS measurement, (b) the astigmatism-corrected beams at the curved multilayer mirror plane which are subsequently forward propagated around the focus, and (c–g) overlapped in-plane amplitude profiles of the 27th harmonic (38.3 nm) for planes denoted in the second row of (b).

The influence of drive laser astigmatism on harmonic beam propagation around the focal region can also be visualized by using overlapping propagation plots (Figure 5). In Figure 5a, b we show overlapped propagated beams that correspond to different astigmatism levels of the drive laser. Here we do not show results for the 23rd harmonic (45 nm), because of the lack of reconstruction for that harmonic from lens tilt equal to θ_opt_ + 1.42°. We notice that, when the drive laser is astigmatic, leading to harmonic beams denoted as red and blue curves, there is a clear presence of lobes that dominate on the *y* direction for negative lens tilt (red curve), and on the *x* direction for positive tilt (blue curve). Also in the diagonal directions, the lobes rotate from one direction to the other as the beam propagates through the focus. In the Supporting Information, we show individual plots of the in-plane overlapped plots of Figure 5c–g, in order to have a clear visualization of the beam properties for each generation condition.

### OAM Modal Purity Analysis

Another important aspect that we can investigate via the spectrally resolved PWFS technique is the modal content and purity of the OAM and how they vary with the generation conditions. For this purpose, we have numerically propagated the wavefronts to the curved multilayer mirror plane and removed the astigmatism caused by the off-axis mirror reflection, as was calculated in the previous section, as well as remaining tip, tilt, and defocus phase terms before calculating the OAM modal content.

**Figure 6:**
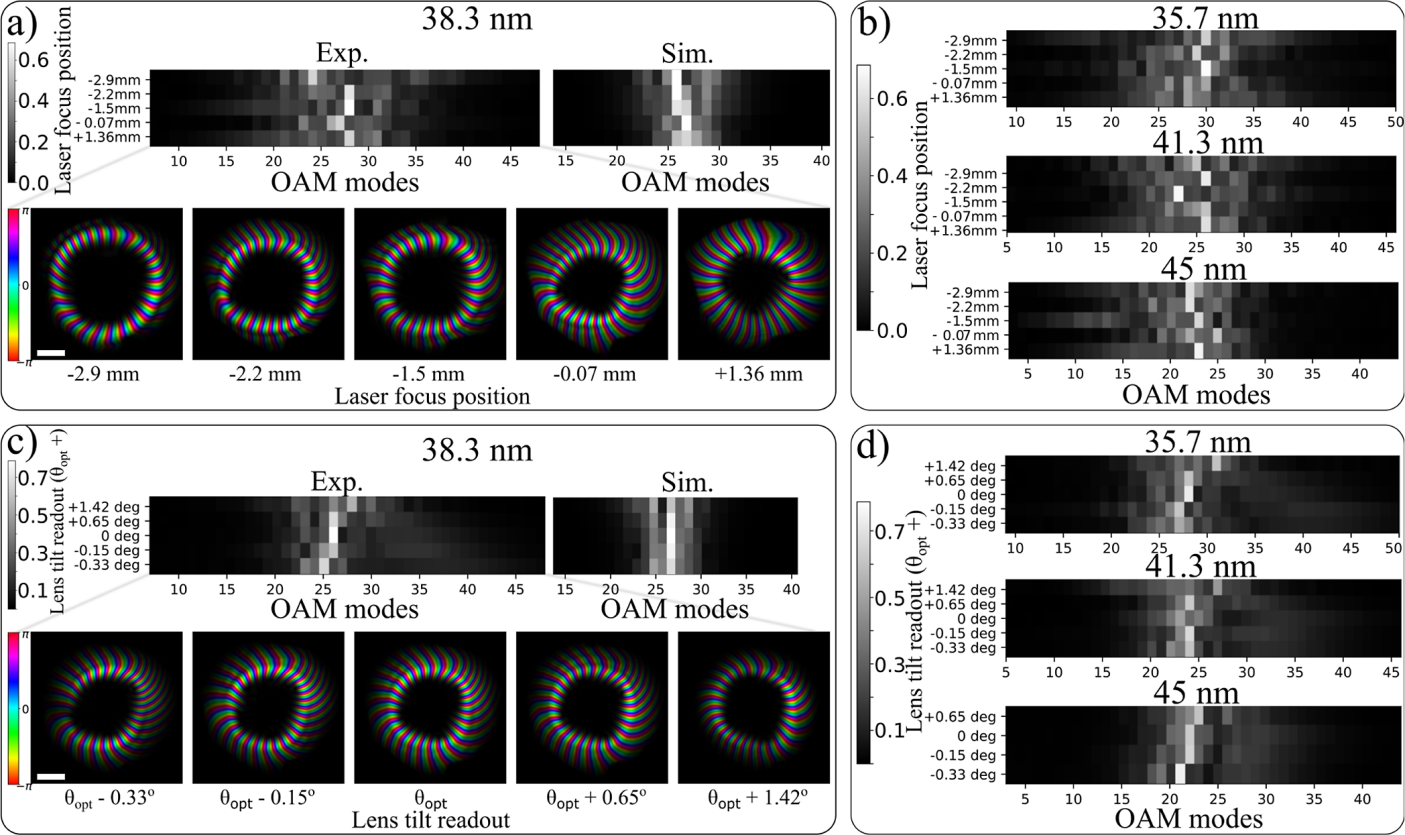
Orbital angular momemtum (OAM) modal content of experimental HHG wavefronts for varying drive laser focus positions with respect to the gas medium. (a) Top: OAM calculated for the 27th harmonic (38.3 nm) and compared to modeled results. Bottom: complex-valued visualization of the 27th harmonic that gave the LG decomposition. (b) OAM content calculated for three harmonic wavelengths, besides the 27th harmonic. (c, d) Same as (a, b) with results from drive laser astigmatism scan. For the beam plots in (a) and (c), brightness indicates amplitude and hue the phase. The scale bars are equal to 3 mm.

As mentioned earlier, the modal content can be calculated either by performing azimuthal FT or through LG decompositions. Due to the slightly elliptic beam profiles, we estimate that the LG decompositions, presented in Figure 6, can give more accurate conclusions, as azimuthal FT along a circular path on an elliptical beam may underestimate the actually present OAMs. However, for comparison reasons, we provide an example of the angular FT method for the case of a circular beam in the Supporting Information, showing very similar results to the LG decomposition. We perform the OAM modal content calculation at the curved multilayer mirror plane for convenience, as it is the reference plane where we correct for the effect of the refocusing optics. However, the LG decomposition would be equivalent at any other plane since the LG modes are solutions to the Helmholtz equation and are propagation-invariant.

Figure 6a displays the experimentally retrieved complex fields of the 27th harmonic for different laser focus positions, together with the OAM modal content from the experimental data and SAR model results. The OAM modal content of the other reconstructed harmonic beams between 35.7 and 45 nm is shown in Figure 6b. The selected laser focus positions are the same five positions with respect to the gas medium, as shown in Figure S2. For the experimental data, we use a truncated basis of LG modes including 5 radial modes, similar to,^34^ and 42 azimuthal modes, whereas for modeled results we use fewer azimuthal modes for computational efficiency. We calculate the basis modes and the coefficients individually for each harmonic beam in our analysis such that the fitting error between the actual beam and its modal description is minimized. As the LG decomposition gives the coefficients *c*_*mn*_ of the complete basis for azimuthal modes *m* and radial modes *n*, we calculate the OAM modal content *C*_*m*_ by summing the coefficients with the same *m* index . In the Supporting Information, we show the complete LG decomposition *c*_*mn*_ for the 27th harmonic and laser focus position −1.5 mm, where we observe that most energy is included in the radial modes with *n* = 0.

The OAM modal content depends on the generation conditions, with the purity being maximized at the laser focus position equal to −1.5 mm. In principle, a broadening of the OAM can be either due to an inhomogeneity in the drive laser amplitude distribution along the vortex ring or due to a deviation of the (unwrapped) phase from a linear behavior in the azimuthal direction. From simulations, the strongest influence is found to result from phase effects, and we attribute the OAM broadening that is shown in Figure 6a, b predominantly to phase aberrations. As discussed earlier, the phase of an HHG wavefront is the sum of the drive-laser-intensity-dependent dipole phase and the drive-laser phase, which both vary significantly with respect to the drive-laser focus position. LG decomposition results of modeled HHG OAM beams also give a broadening of the OAM modal content for certain laser focus positions. The simulation results in Figure 6a show qualitative agreement with the experimental data, but the variations with laser focus position are not fully reproduced.

The dependence of the OAM modal content on drive laser astigmatism is shown in Figure 6c, d. For increasing astigmatism, we observe a broadening of the OAM distribution. In addition, there appears to be a small linear increase in the mean OAM value as a function of lens tilt. The OAM broadening, which is qualitatively reproduced by the simulations, is related to the impurity that is injected into pure LG_*q*_, with *q* being the harmonic order when an astigmatism phase term and an amplitude modulation are added to an azimuthally symmetric beam. The astigmatism phase term leads to local deviations from the linear azimuthal phase ramp for an ideal LG_*q*_ mode, resulting in variations in the OAM. However, from symmetry considerations, any effect of the lens tilt should be independent of the sign, as a positive and negative tilt gives rise to the same induced astigmatism. SAR simulations of the drive laser beam as a pure LG_1,0_ mode with added astigmatism confirm that expectation. Therefore, the observed linear trend is likely due to other experimental uncertainties resulting from changes in lens alignment, which may influence both the fundamental wavefront and the HHG process. Determining the origin of this OAM variation will require further study.

### Synthesis of Attosecond Pulse Trains

The spectrally resolved amplitude and phase reconstruction of multiple high harmonics enables the reconstruction of the attosecond pulse train (assuming the harmonics are phase-locked) by Fourier-transforming the reconstructed frequency-domain fields to the time domain. For ideal drive laser conditions, the attosecond pulse is expected to form a double-sided helix.^19,43^ However, the influence of the dipole phase and drive laser aberrations on the HHG wavefronts can distort the double helix structure. In the Supporting Information, we show synthesized attosecond pulse trains from the reconstructed harmonic wavefronts under certain assumptions for the spectral phase that would correspond to Fourier-transform limited pulses. However, a conclusive analysis would require a measurement that is sensitive to the relative phase between the harmonics.^65^

## Conclusions

In conclusion, we utilized the spectrally resolved PWFS method for characterizing HHG OAM beams for different generation conditions. We showed how changing the laser focus position with respect to the gas jet leads to different far-field HHG beam profiles. We attributed this behavior to the dipole phase associated with the short electron trajectory. Furthermore, any astigmatism in the drive laser is upconverted to the high harmonics such that even a slight astigmatism of the drive laser leads to the emergence of bright and dark areas along the doughnut-shaped beam. Except for observations related to the amplitude of the HHG beams, we could investigate more properties of the multiwavelength vortex beams under the assumption that we know precisely the astigmatism induced by the refocusing optics. The influence of the laser focus position and the laser aberrations on the HHG beam properties around the focal region could be characterized in this way. By performing LG decompositions on the beams for each generation condition, we found that these differences can be also associated with the broadening of the OAM modal content of the beams. Finally, we proposed a simplified approach to reconstruct the attosecond pulse train for different generation conditions, assuming prior knowledge of the spectral phase. In the Supporting Information, we show results from this approach, where we observed how impurities in the OAM modal content distort the double-helix light spring structure that is theoretically predicted in previous studies.^19,43^ In particular, aberrations in the harmonic fields are found to lead to a modification of the double helix structure, in both intensity and position.

Overall, the PWFS method is a high-fidelity technique for characterizing multiple HHG wavefronts with high resolution from a single measurement, and in this work, we have extended this capability to highly structured XUV beams carrying high topological charge. The complex-valued reconstructions of the wavefronts enable numerical back-propagation to the generation plane, where we can study the upconversion mechanisms and experimentally validate theoretical models about the generation of such XUV vortex beams.

An interesting follow-up of this work could be on performing PWFS measurements for more complex multiwavelength vortex HHG beams, such as the beams demonstrated in previous studies.^26,29,30,33,36,37^ In these cases, the topological charge values of the harmonics are relatively unknown, and we would need to implement unconstrained ptychographic reconstructions in terms of the topological charge values. Preliminary PWFS measurements with single-wavelength HHG vortex beams, not presented in this work, showed that for this less complicated parameter space, the topological charge was reconstructed accurately without any prior knowledge requirement on the topological charge. Furthermore, for multiwavelength Gaussian beam PWFS, we have demonstrated high-fidelity unconstrained wavefront reconstructions by increasing the overlap factor between adjacent scan positions to a value above 90%.^53^ Therefore, we believe that PWFS on more complex multiwavelength vortex HHG beams is possible but would require many scan positions in order to satisfy a high overlap factor, and possibly high exposure times so that the recorded diffraction patterns have high SNR even for the first- and second-order diffraction signal. The design and implementation of such a measurement require a very stable HHG beam in terms of long-term beam drifts and intensity fluctuations.

This work on HHG vortex beams generated by a single vortex drive beam offers insight into how critical parameters such as drive laser focus and aberrations can be in achieving desired properties for the attosecond pulse. We believe that this work is a first step toward more efficient design and experimental implementation with attosecond OAM beams.

## Data Availability

All data underlying the results of this paper may be obtained from the authors upon reasonable request.

## Supporting Information Available

(I) Ptychographic reconstruction without constraint on topological charges; (II) HHG far field beam amplitudes for varying generation conditions; (III) Simulated long trajectory contributions; (IV) propagation of HHG OAM beams; (V) calculation of OAM modal content; (VI) synthesis of attosecond pulse trains from ptychographic reconstructions (VII) Figures S1–S10 that complement the findings in the main text; and (VIII) animated propagation of an astigmatism-corrected OAM beam through the focus (PDF)
Propagation beam along focus (MP4)
