# Supplementary material for: Generation Dynamics of Broadband Extreme Ultraviolet Vortex Beams
Source: ACS Photonics. 2025 Feb 28;12(3):1638–49. doi: 10.1021/acsphotonics.4c02516 (PMC11926965; doi:10.1021/acsphotonics.4c02516)
Supplement: Supplementary file 1 — ph4c02516_si_001.pdf [file ph4c02516_si_001.pdf]

Generation dynamics of broadband extreme ultraviolet vortex beams - Supplementary information

Antonios Pelekanidis,[†,‡] Fengling Zhang,[†,‡] Kjeld S. E. Eikema,[†,‡] and Stefan Witte*,[†,‡]

†*Advanced Research Center for Nanolithography, Science Park 106, 1098 XG, Amsterdam, The Netherlands*

‡*Department of Physics and Astronomy, Vrije Universiteit, De Boelelaan 1105, 1081 HV Amsterdam, The Netherlands*

E-mail: witte@arcnl.nl

Ptychographic reconstruction without constraint on topological charges

As mentioned in the "Materials and Methods" section of the main text, the resulting topological charges of the reconstructed harmonic wavefronts may vary between independent reconstructions, due to the low overlap between scan positions and the complexity of the beams. In Fig. S1a) we show example reconstructions of wavefronts with the correct and incorrect topological charges. The correct topological charges were achieved via imposing constraints during the reconstruction process. We observe that the intensity profile of the beams at the ptychographic wavefront sensor (PWFS) plane exhibits large areas of low intensity and fast fringe-like features at the vicinity of the bright lobes. This structure of

the beams renders the wavefront reconstruction very sensitive to the the phase, which is undefined when intensity values are (near) zero.

In Fig. S1b) we show backpropagated beams to a plane where the beam profile has a doughnut shape and the topological charge can be calculated via integration following a closed loop with non-negligible intensity values. Overlapping the intensity plots between correct and incorrect reconstructions reveals that even a difference of 2 between topological charges does not affect strongly the beam propagation behavior. Therefore, we consider that a deviation of ± 2 in the resulting topological charge of the reconstructed beams is to be expected and a constraint on the topological charge during the reconstruction was necessary to further constrain the reconstruction process and lead to a unique solution.

HHG far field beam amplitudes for varying generation conditions

Laser focus position scan

To complement Fig. 2a) of the main manuscript in which we showed data for 3 lens positions, Fig. S2 presents results from the complete dataset of 5 lens positions that aimed to investigate how the drive laser focus position with respect to the gas jet affects the HHG beam amplitudes in the far field. Experimental results in Fig. S2a) can be compared with simulation results (Fig. S2b)). We observe a close agreement between simulations and experiment, especially with related to the rim thickness and the presence of secondary concentric grids.

Drive laser astigmatism scan

Complementing Fig. 2c), Fig. S3 displays results from the complete dataset of 5 lens tilt positions that lead to different astigmatism levels of the drive laser. The experimental

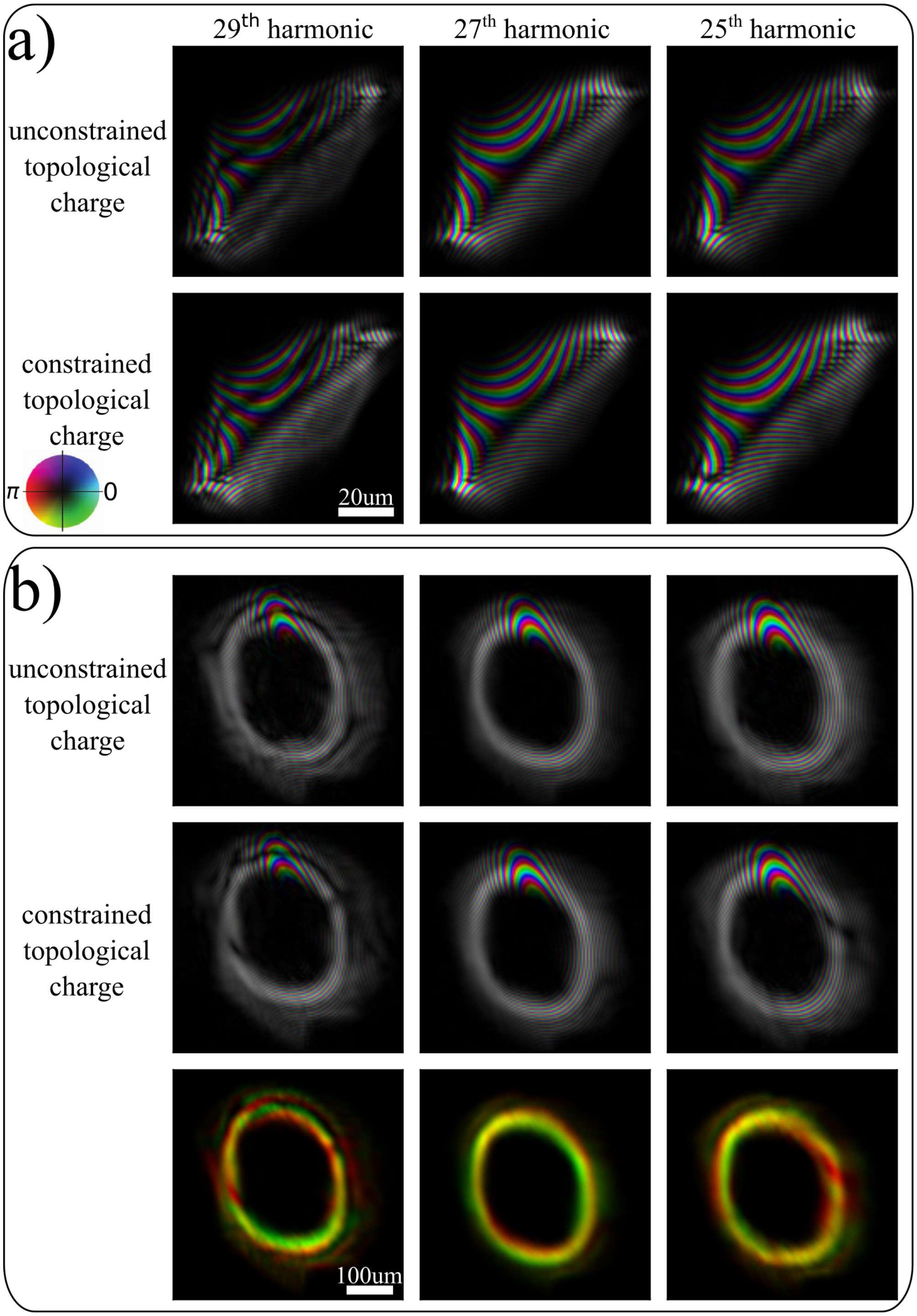

Figure S 1: Reconstructed beams with and without imposing constraints on the topological charges. a) Complex-valued plots of the reconstructed beams for three harmonics ranging from 35.7 nm (29th harmonic) to 41.3 nm (25th harmonic) at the PWFS plane. All figures share the same scale bar. b) Propagated beams from a) to a plane 10 mm upstream, where the beam shape looks more like a doughnut. Topological charges are calculated as (29,27,25) from the constrained reconstruction, and (27,25,23) from the unconstrained reconstruction, for harmonics (29,27,25) respectively. The last row shows overlapped intensity plots of the uncostrained (red) and constrained (green) results. All figures share the same scale bar.

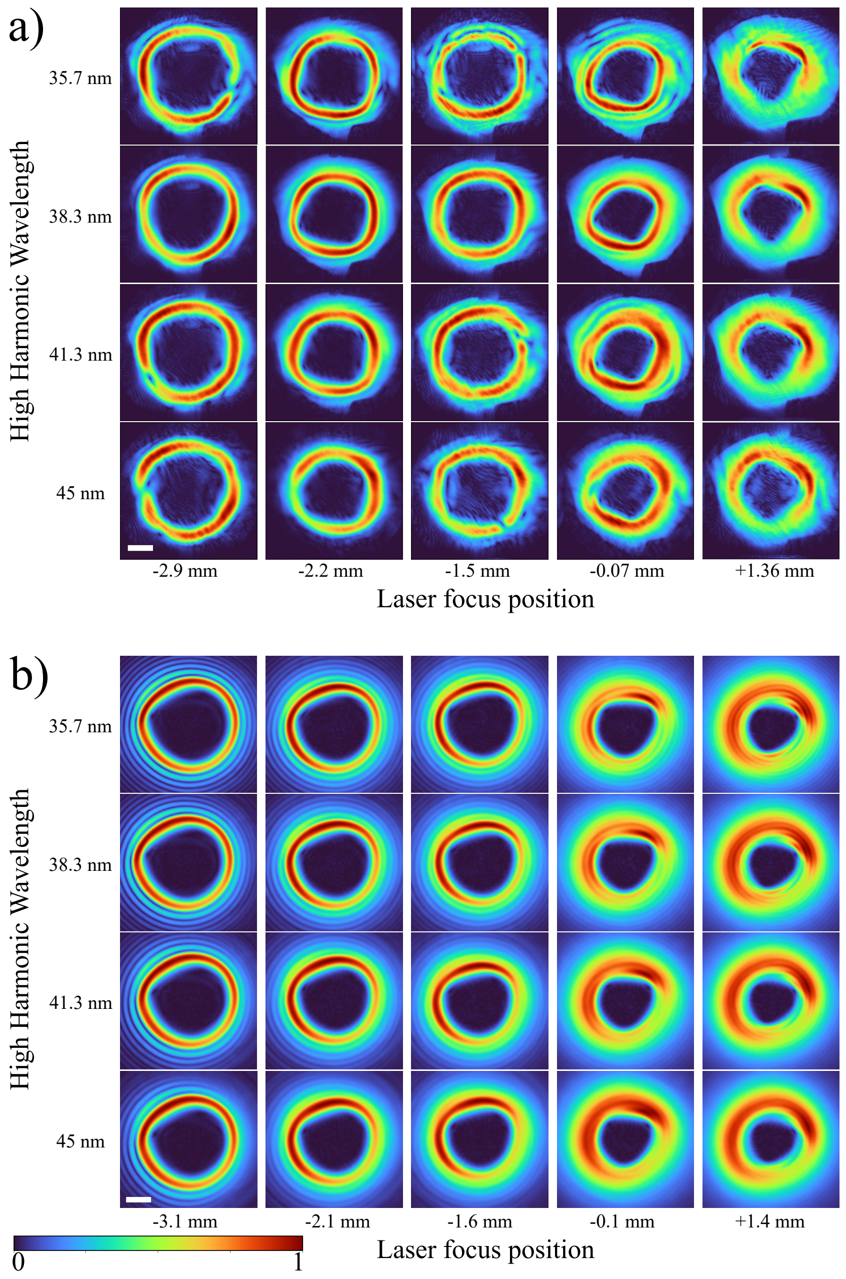

Figure S 2: Reconstructed beam amplitudes for four harmonics ranging from 35.7 nm (29th harmonic) to 45 nm (23rd harmonic) at the curved multilayer mirror plane for various laser focus positions with respect to the gas medium. Positive (negative) laser focus positions means that the laser focus is downstream (upstream) of the gas jet position. a) Reconstructed beams from experimental data, b) modelled beams. Scale bars are equal to 3 mm.

results (Fig. S3a)) can be compared with simulation results (Fig. S3b)).

Laser focus position scan for ideal drive beam

In the main text we have shown that the exact drive beam properties influence the far-field HHG beam intensity profiles, with secondary rings appearing when the drive laser focus position is upstream of the generation plane. We have also shown that the presence of the rings can be explained and modelled considering only short trajectory contributions within the HHG process. In this section we repeat the simulation of generation and numerical propagation of HHG beams for a drive beam with perfect circular symmetry and without aberrations.

In order to be consistent with other parameters, such as drive beam size at the (varying) generation plane, we simulate the drive beam in the same way as we did to generate the modelled results of Figs. 2, S2 and S3. We started with the reconstructed IR Gaussian wavefront from the fundamental ptychography reconstruction, which we propagated numerically to the lens plane. At the lens plane we fitted a circularly symmetric Gaussian profile to the beam intensity, removed the existing phase and added manually a quadratic phase that corresponds to an ideal lens with focal length equal to 30 cm.

The results of the far-field HHG beam amplitudes for varying drive laser focus positions is shown in Fig. S4 for the 27th harmonic (38.3 nm) as an example. The intensity profiles for different harmonics in the examined range of harmonic orders (23-29) are slightly different but exhibit the same trend. We observe a single ring in the far field, with a varying thickness for different laser focus positions. These results are in good agreement with other reported theoretical results.[1] Therefore, we can conclude that the appearance of the multiple rings is due to the specific drive beam properties.

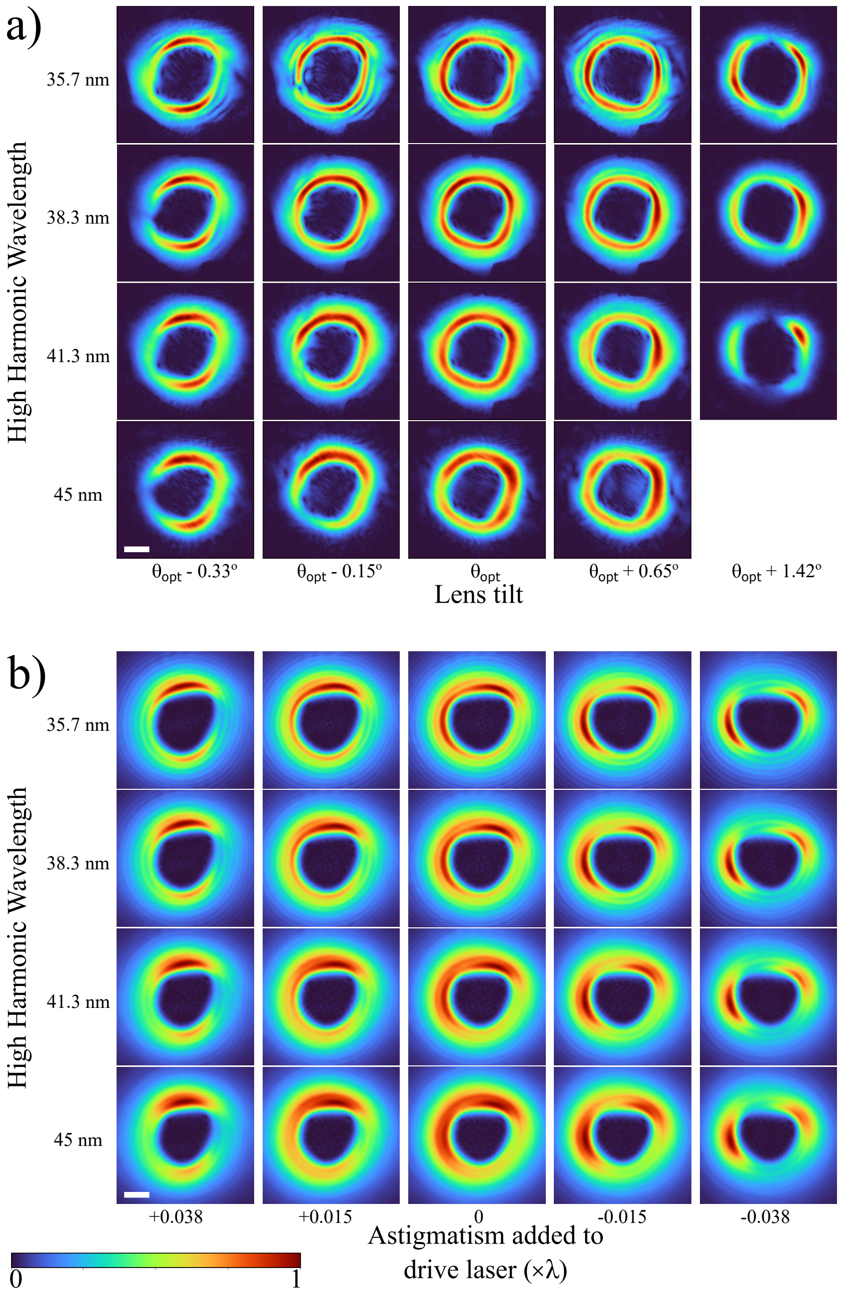

Figure S 3: Reconstructed beam amplitudes at curved multilayer mirror plane for varying tilts of the focusing lens of the drive laser. a) Reconstructed beams from experimental data, b) modelled beams. In a) the reconstructed beam at 45 nm wavelength and $\theta_{opt} + 1.42°$ tilt angle is missing because the reconstruction was not trustworthy due to low signal within the beam profile. Scale bars are equal to 3 mm.

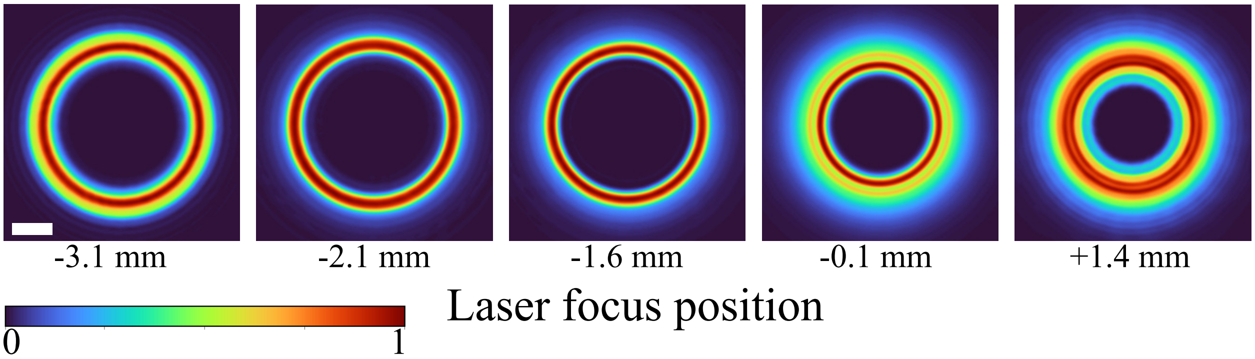

Figure S 4: Modelled beam amplitudes for the 27th harmonic (38.3 nm) at the curved multilayer mirror plane for various laser focus positions with respect to the gas medium. The drive beam is simulated with an ideal circularly symmetric intensity profile and a non-aberrated phase. Here we consider only short trajectory contributions. Scale bar is equal to 3 mm.

Simulated long trajectory contributions

To complement the analysis of the modelled high harmonic wavefronts, we present the simulated wavefronts that would correspond to the long trajectory contributions. As mentioned in the main text, we use the single atom response (SAR)[2,3] model that gives the following expressions for the amplitude A and the phase Φ of each high harmonic q in the plateau region for the long trajectories:

$$A_q(x,y) = A_f(x,y)^p$$
$$\Phi_q(x,y) = q\phi(x,y) + \Phi_{l,q}(x,y)$$
$$\Phi_{l,q}(x,y) = \alpha_l I(x,y) + \frac{\gamma_l}{I(x,y)}(q\omega - \Omega_p)^2 \ + \ const.$$

The values of the constant factors are adjusted from[3] for a drive laser wavelength equal to 1030 nm, $\alpha_l = -5.08 \times 10^{-13}$ W^{-1}cm^2, $\gamma_l = -0.685 \times 10^{-18}$ s^{2}Wcm^{-2}. The drive beam parameters and identical to the ones used in the main text to calculate the short trajectory contributions (pulse energy $E \approx$1.3 mJ, and pulse duration $\Delta t = 45$ fs). Example for the 27th harmonic (38.3 nm) of the complex-valued beam profile at the gas jet plane for varying drive laser focus position is shown at Fig. S5. All harmonics in the examined range of harmonic

orders 23-29 have a similar behavior. We observe a very strong quadratic behaviour for the phase in radial direction, which we have verified that is caused by the term $\alpha_l I(x,y)$. Upon propagation to the far field, this beam does not remain well-defined and we cannot detect it.

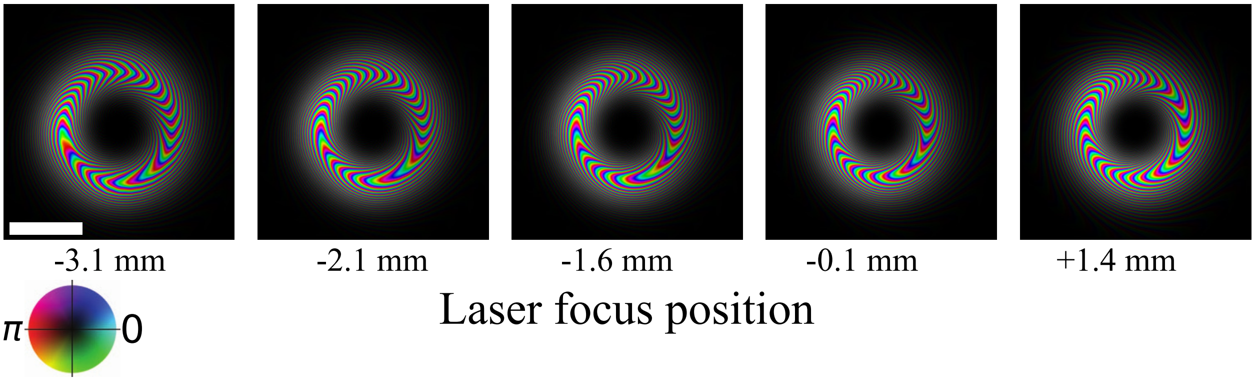

Figure S 5: Modelled long trajectory contributions of the 27th harmonic (38.3 nm) at the generation plane, for various laser focus positions with respect to the gas medium. Scale bar is equal to 50 µm.

Propagation of HHG OAM beams

Diagonal cross-sections of propagation plots

Ptychographic reconstruction of the complex fields of individual harmonics enables numerical propagation of the beams. In the main text we have shown horizontal and vertical cross-sections of the numerical propagation for the reconstructed and astigmatism-corrected HHG wavefronts. However, since the studied beams exhibit strong asymmetries azimuthally, we also show diagonal cuts for a more complete view of the beam propagation behavior in Fig. S6.

Effect of dipole phase on rim thickness

From overlapping propagation plots of HHG beams for different laser focus positions, we observed in the main text that there is a local peak in the rim thickness of which the position

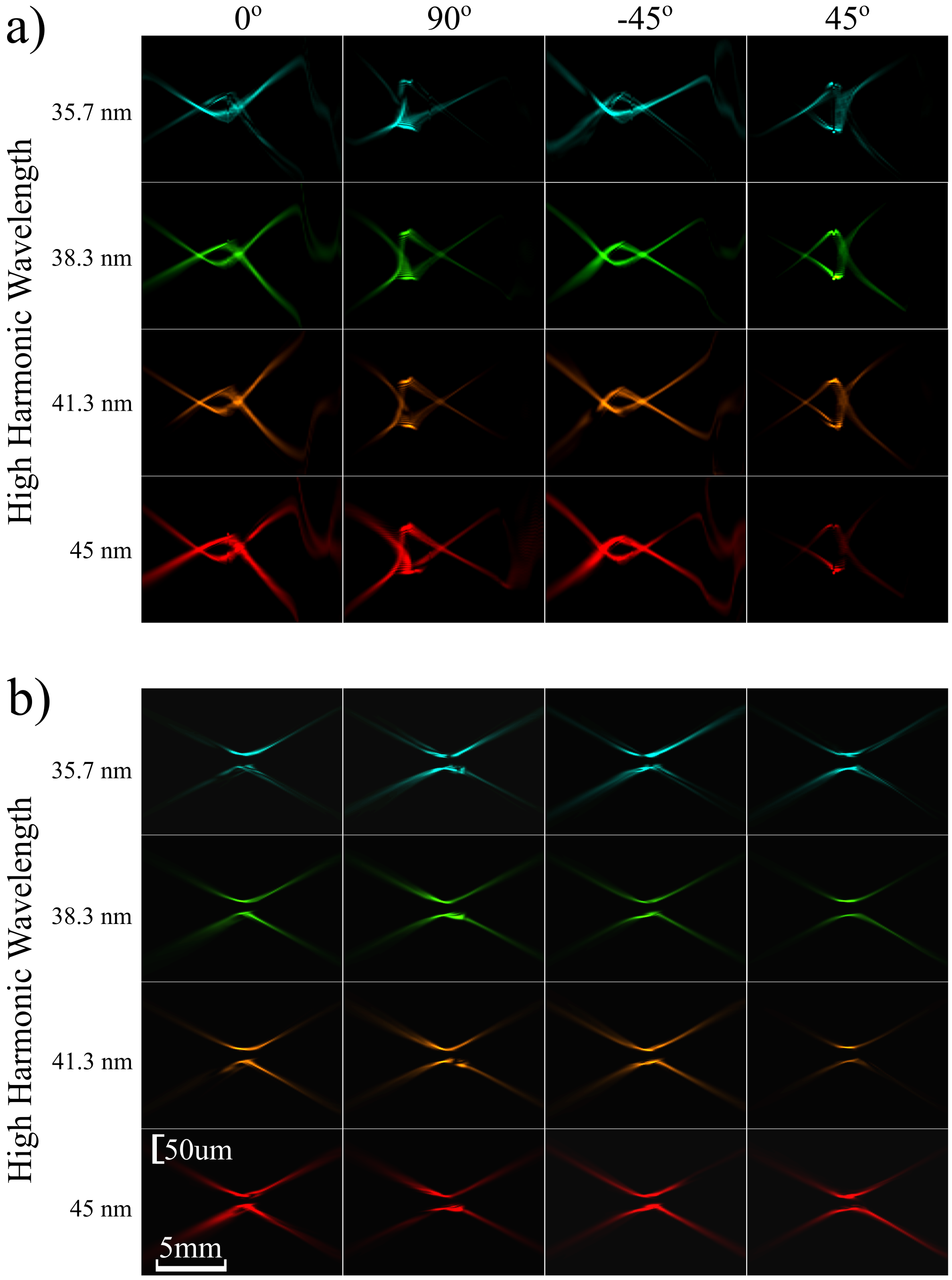

Figure S 6: Propagation of harmonic beams along the focal plane after refocusing. a,b) Cross sections along different cuts (horizontal, vertical and diagonal) for a) the reconstructed wavefronts from the PWFS measurement, b) the astigmatism-corrected beams at the curved multilayer mirror plane which are subsequently forward propagated around the focus.

varies with respect to the focal plane. From the simulations we could confirm that this peak is located at the generation plane. Figure S7a) shows the effect of the dipole phase on the rim for the 27th harmonic (38.3 nm), where we observe a subtle, but observable effect of the dipole phase. Specifically, we notice that the falling edge of that local maximum in the rim shifts slightly downstream. This finding supports our explanation in the main text that the dipole phase, which has a profile inversely proportional to the drive laser intensity, creates local divergent wavefronts that lead to expansion of the rim. This tendency to expand is eventually counteracted by an opposite effect caused by the fact that the present LG modes in the beam move out of phase upon propagation. The results shown in Fig. S7a), as well

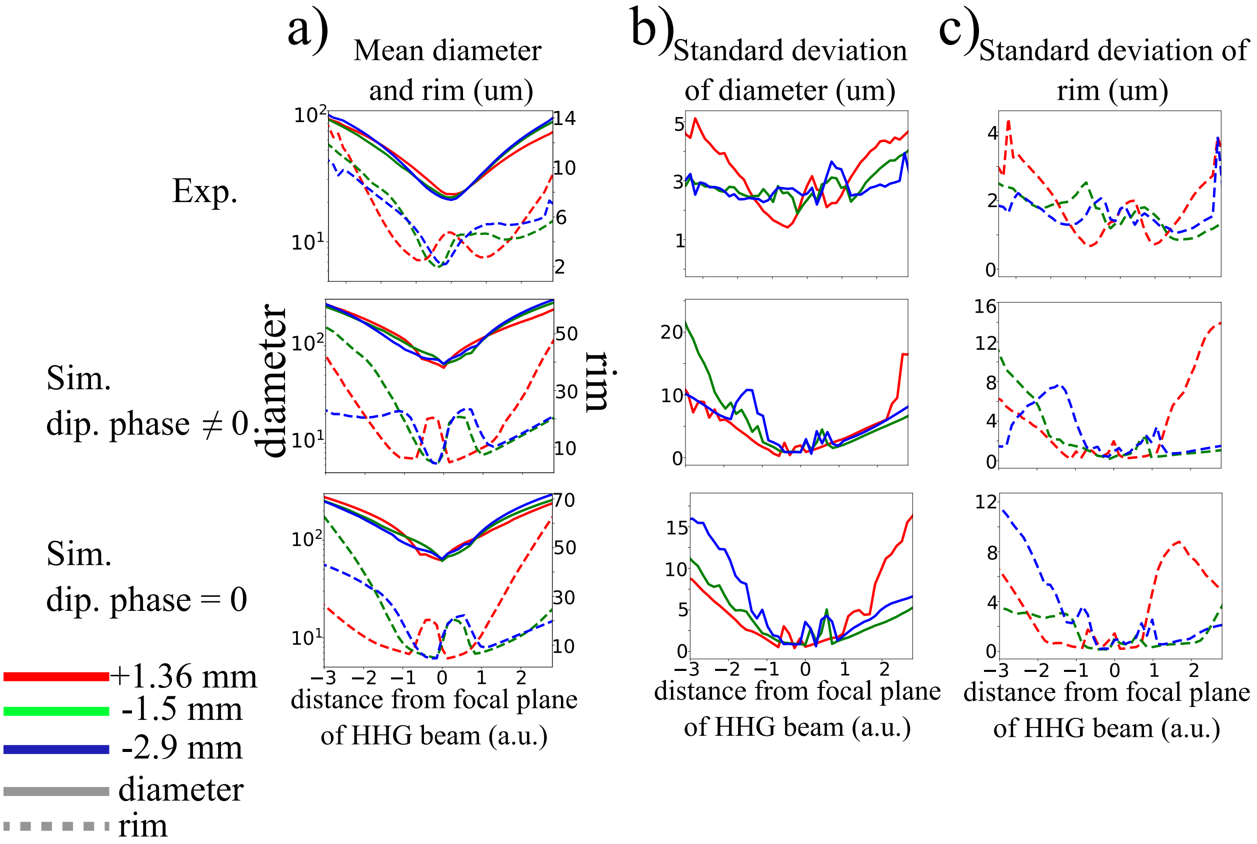

Figure S 7: Experimental and model results for the variation of diameter and rim for the 27th harmonic (38.3 nm), with comparison of the model with and without the dipole phase contribution.

as in the main text, are averaged plots over all azimuthal angles. Due to asymmetries in the HHG OAM beam profiles, both diameter and rim can vary for different azimuthal angles.

For completeness, in Figs. S7b) and S7c) we show standard deviation plots for the diameter and rim respectively, of the results of Fig. S7a). We observe that the standard deviation is always considerable smaller than the mean value for all datasets and propagation positions.

Propagation of astigmatic HHG OAM beams

In the main text we show overlapped in-plane plots of HHG OAM beams for different drive laser astigmatism levels. In Fig. S8 we separate the three color channels, so that it is clear how the 27th harmonic (38.3 nm) beam looks for different laser astigmatism conditions, as it propagates through the focus.

Calculation of OAM modal content

The OAM modal content of an OAM beam can be determined either by a Laguerre-Gaussian (LG) decomposition of the beam or by performing 1D azimuthal Fourier Transform (FT) along the beam. In the analysis presented in the main text, we used LG decomposition as the experimental beams were not perfectly circular and an azimuthal FT would potentially not be accurate. However, for a circular beam, which occurred when we minimized the drive laser astigmatism, the results from LG decomposition and FT are quite similar, as shown in Fig. S9. We have performed azimuthal FT along 5 rings with varying radius, in a range where the beam intensity is significant and present the results in Fig. S9c). The results are in very close agreement with the LG decomposition results shown in Fig. 6a) of the main text for the same beam, which corresponds to laser focus position equal to -1.5 mm.

Furthermore, in Fig. S9b) we show the complete LG decomposition of the same beam, with coefficients for all radial and azimuthal modes. We observe that there is a dominant LG mode with zero radial nodes, but higher radial modes are also present. Assuming the LG coefficients are preserved over their propagation from the gas jet to the far field, the presence of many radial modes with a dominant zeroth order radial mode explains why the

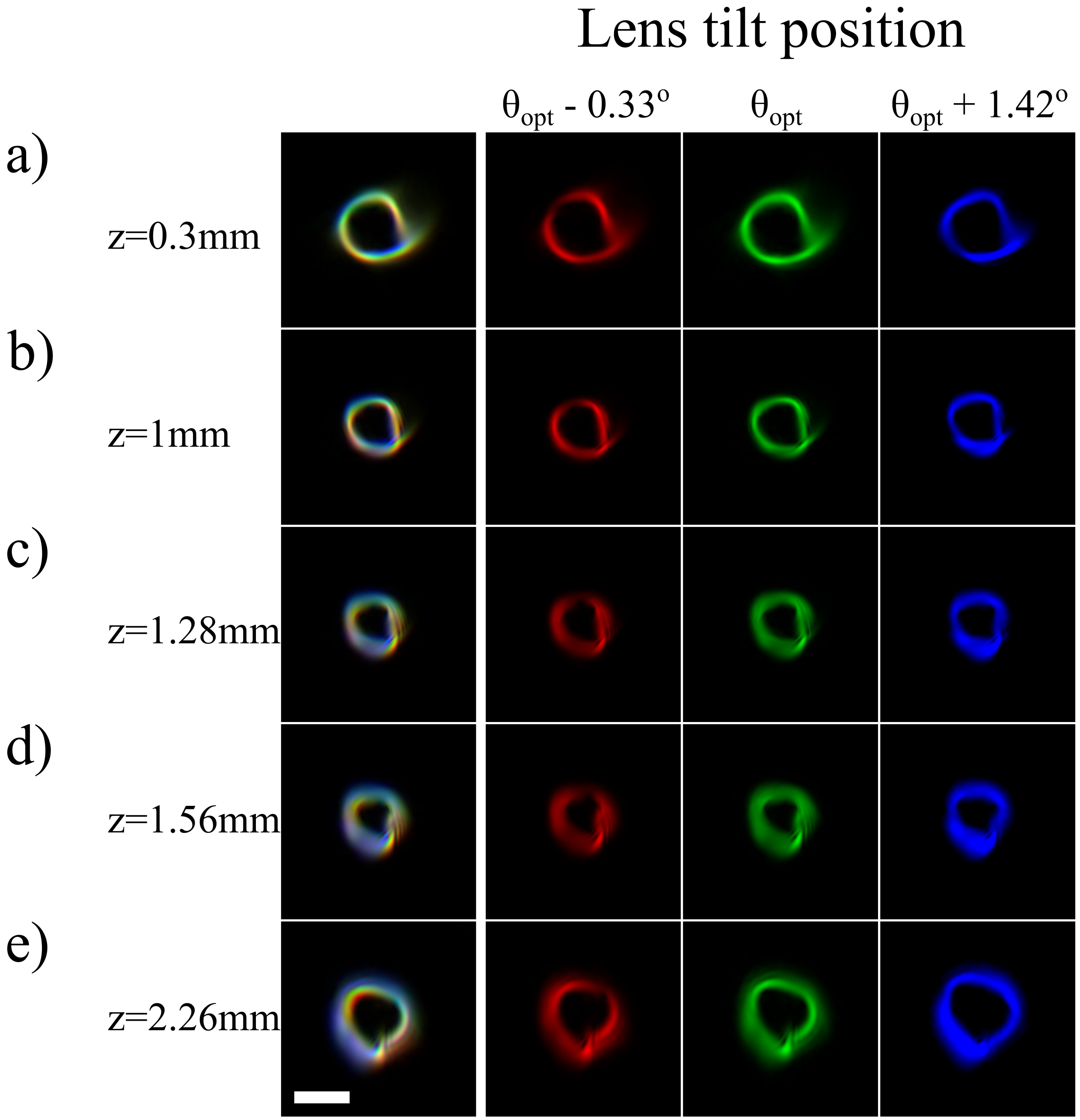

Figure S 8: Decomposition of the overlapped beam plots of Fig. 5c-g) of the main text into separate color channels. Note that the $\theta_{opt} + 1.42°$ results are shown in a reduced dynamic range for better visibility. The scale bar is equal to 30 µm.

rim exhibits the behavior shown in Fig. S7a).

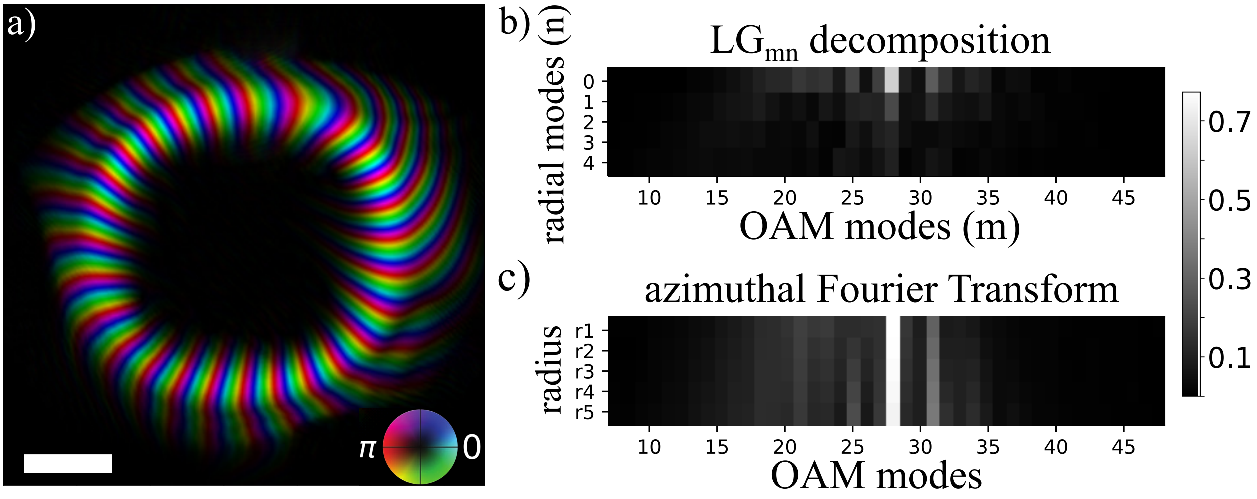

Figure S 9: Orbital angular momemtum (OAM) modal content of the 27th harmonic (38.3 nm) for drive laser focus position equal to -1.5 mm. a) beam amplitude and phase, b) complete c_{mn} LG modal decomposition into azimuthal and radial modes, c) calculated OAM content via azimuthal Fourier Transform on varying radii. The scale bar in a) is equal to 3 mm.

Synthesis of attosecond pulse trains from ptychographic reconstructions

In this analysis we are interested in studying how the generation conditions affect the generated attosecond pulse, thus we remove all known effects of the multilayer mirrors, namely the shaping of the spectral weights and the astigmatism induced by the spherical multilayer mirror.

The ptychographic reconstruction algorithm treats the wavefronts as independent incoherent modes and is insensitive to the relative phase between the harmonic beams. Therefore, we can only assume phase coherence between the harmonics, and numerically fix the phase of all harmonic beams at a manually selected bright part of the polychromatic beam at time $t = 0$. This assumption ensures that all harmonics constructively interfere to create one of the two helices. The resulting spatial and temporal intensity profiles are shown for different

laser focus positions in Fig. S10a), and for different amounts of astigmatism in Fig. S10b). We also show examples of the temporal evolution of the attosecond pulse train over an extended period of 6 fs for laser focus position equal to +1.36 mm (Fig. S10a)) and drive laser astigmatism from lens tilt equal to $\theta_{opt} + 0.65°$ (Fig. S10b)). Here we have assumed that we have a periodic signal with a slight amplitude modulation that follows the Gaussian temporal shape of the drive laser. From the comparisons between different generation conditions, we observe that the generation conditions only have limited effect on the temporal shape of the attosecond pulse train, although the broadened OAM modal content of the harmonic wavefronts lead to pre- and post-pulses that are sensitive to the laser focus position.

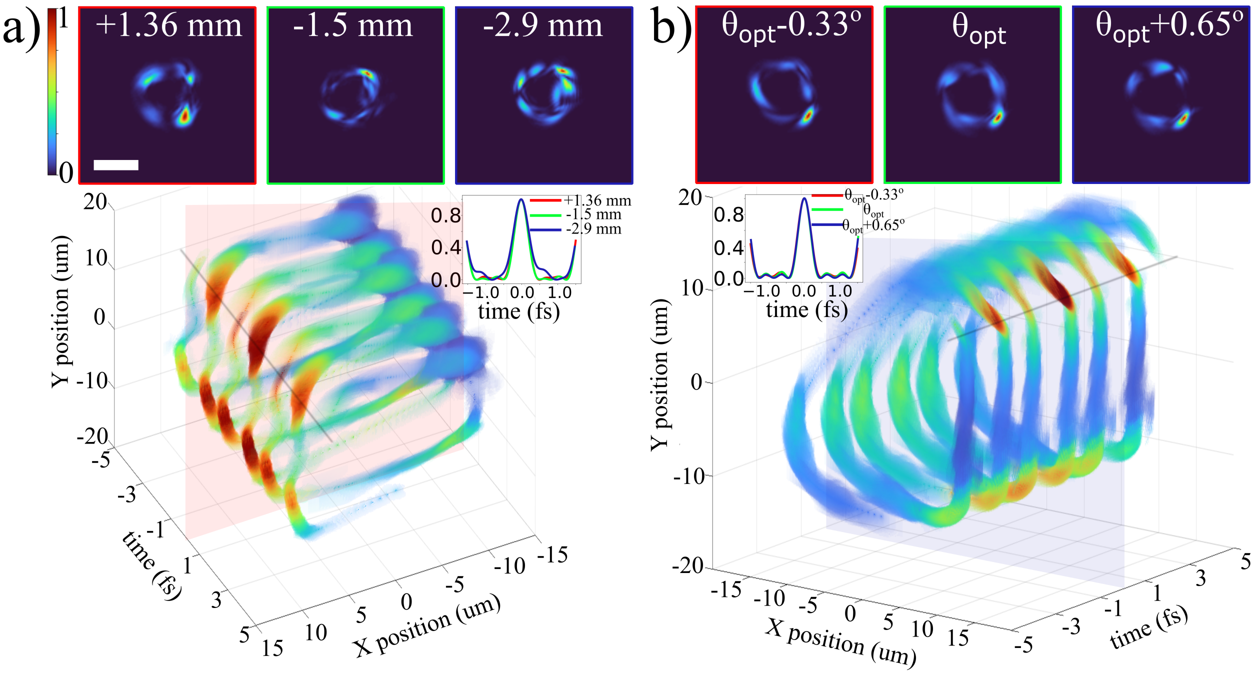

Figure S 10: Comparison of attosecond pulse trains for a) different drive laser focus positions, and b) different drive laser astigmatism levels, expressed as lens tilt positions of the focusing lens to the gas jet. Top: spatial intensity profiles at time $t = 0$, bottom: Temporal evolution of the high-harmonic signal for a) laser focus position +1.36 mm and b) lens tilt position $\theta_{opt} + 0.65°$. The colored planes indicate the $t = 0$ plane. The insets show the comparison of the temporal evolution at a specific point in space with high intensity at $t = 0$, as indicated with the solid gray line in the 3D plots. Scale bar is shared among all spatial intensity profile figures and is equal to 20 µm.

However, the spatial properties of the pulse train are more significantly affected by the generation conditions. From the spatial properties of the pulse at $t = 0$ we can distinguish

the manually fixed bright spot, which is located either at the top right part or at the bottom right part of the beams. Interestingly, at the opposite side of the ring, the second helix appears weaker, which indicates that matching the phases for the one helix does not ensure perfectly constructive interference at the second helix in the presence of aberrations in the harmonic wavefronts. Instead, there are secondary bright parts spread at various azimuthal angles, which appear as pre- and post-pulses in the temporal plots of Fig. S10. Finally, in Fig. S10b) we notice that the astigmatism of the drive laser leads to either clockwise or counterclockwise shift of the second helix compared to the stigmatic case, depending on the positive or negative tilt angle of the focusing lens. These results give an indication of the possible spatiotemporal structure of the attosecond pulse train that can be synthesized with the experimentally observed harmonic fields.

References

(1) Hernández-García, C.; San Román, J.; Plaja, L.; Picón, A. Quantum-path signatures in attosecond helical beams driven by optical vortices. *New Journal of Physics* **2015**, *17*, 093029.

(2) Guo, C.; others Phase control of attosecond pulses in a train. *Journal of Physics B: Atomic, Molecular and Optical Physics* **2018**, *51*, 034006.

(3) Wikmark, H.; Guo, C.; Vogelsang, J.; Smorenburg, P. W.; Coudert-Alteirac, H.; Lahl, J.; Peschel, J.; Rudawski, P.; Dacasa, H.; Carlström, S.; others Spatiotemporal coupling of attosecond pulses. *Proceedings of the National Academy of Sciences* **2019**, *116*, 4779–4787.
